# Mutational Analysis of the Putative Anti-Müllerian Hormone (AMH) Binding Interface on its Type II Receptor, AMHR2

**DOI:** 10.1210/endocr/bqaa066

**Published:** 2020-04-25

**Authors:** Kaitlin N Hart, David Pépin, Magdalena Czepnik, Patricia K Donahoe, Thomas B Thompson

**Affiliations:** 1 Department of Pharmacology and Systems Physiology, University of Cincinnati, Cincinnati, OH, USA; 2 Department of Molecular Genetics, Biochemistry, and Microbiology, University of Cincinnati, Cincinnati, OH, USA; 3 Department of Surgery, Massachusetts General Hospital, Harvard Medical School, Boston, MA, USA; 4 Pediatric Surgical Research Laboratories, Massachusetts General Hospital, Boston, MA, USA

**Keywords:** anti-Müllerian Hormone (AMH), AMHR2, MISR2, ligand-receptor interactions, transforming growth factor beta (TGF-β), mutagenesis

## Abstract

Anti-Müllerian hormone (AMH) or Müllerian inhibiting substance is a unique member of the TGF-β family responsible for development and differentiation of the reproductive system. AMH signals through its own dedicated type II receptor, anti-Müllerian hormone receptor type II (AMHR2), providing an exclusive ligand-receptor pair within the broader TGF-β family. In this study, we used previous structural information to derive a model of AMH bound to AMHR2 to guide mutagenesis studies to identify receptor residues important for AMH signaling. Nonconserved mutations were introduced in AMHR2 and characterized in an AMH-responsive cell-based luciferase assay and native PAGE. Collectively, our results identified several residues important for AMH signaling within the putative ligand binding interface of AMHR2. Our results show that AMH engages AMHR2 at a similar interface to how activin and BMP class ligands bind the type II receptor, ACVR2B; however, there are significant molecular differences at the ligand interface of these 2 receptors, where ACVR2B is mostly hydrophobic and AMHR2 is predominately charged. Overall, this study shows that although the location of ligand binding on the receptor is similar to ACVR2A, ACVR2B, and BMPR2; AMHR2 uses unique ligand-receptor interactions to impart specificity for AMH.

Anti-Müllerian hormone (AMH) or Müllerian inhibiting substance (MIS) is a secreted protein hormone known for its critical role in the development of the reproductive system and sex determination. In males, AMH is expressed early in the Sertoli cells of the testes, where it functions to cause regression of the Müllerian duct during fetal development and, subsequently along with testosterone, to differentiate the Wolffian duct into male reproductive organs (1-5). In women, AMH is expressed by the granulosa cells of the ovary, where it is responsible for regulation of various aspects of folliculogenesis (3, 6, 7). Diagnostically, serum AMH levels are now routinely used clinically as a marker for measuring ovarian reserve in females (8, 9). Several studies have shown that loss of function, likely pathogenic mutations in either AMH or its receptor anti-Müllerian hormone receptor type II (AMHR2), are associated with the development of persistent Müllerian duct syndrome (PMDS) in humans and other mammals (10-12). PMDS is a form of pseudohermaphroditism characterized by the presence of Müllerian duct derivatives, such as a uterus and Fallopian tubes, in males (11).

More recently, studies have suggested that AMH signaling may play a role in androgen regulation in polycystic ovarian syndrome (PCOS) (13, 14). Loss-of-function mutations in AMH and AMHR2 have been identified in PCOS patients, which could result in dysregulation of enzymes involved in the synthesis of testosterone, leading to a hyperandrogenic state. Alternatively, other studies have shown that AMH serum levels are often elevated in women with PCOS and that AMH can inhibit enzymes important for androgen metabolism (15-18). As such, an imbalance—increased or decreased—of AMH signaling may explain the increase in androgen levels found in PCOS patients. However, further research is needed to investigate the role of AMH within PCOS.

AMH is a member of the TGF-β family, as characterized by Cate et al. in 1986. This family consists of more than 30 unique signaling molecules that are subdivided into 3 major ligand classes (bone morphogenetic proteins [BMPs], TGF-βs, and activins) (19, 20). Ligands of the family are typically covalently linked dimers with a conserved propeller-like shape from a top view (Fig. 1A, top right) and a butterfly shape from a side view (Fig. 1A, bottom right) (20, 21). TGF-β ligands signal by assembling 2 type I and 2 type II serine/threonine kinase receptors of which there are 5 type II receptors, ACVR2A (ActRIIA), ACVR2B (ActRIIB), BMPR2, TGFβR2, and AMHR2 (MISR2), and 7 type I receptors, ACVRL1 (Alk1), ACVR1 (Alk2), BMPR1A (Alk3), ACVR1B (Alk4), TGFβR1 (Alk5), BMPR1B (Alk6), and ACVR1C (Alk7) (20). The assembled ligand:receptor complex allows the constitutively active type II receptor to activate the type I receptor, which in turn phosphorylates intracellular Smad proteins that serve as transcription factors. In general, ligands produce a signal through 1 of 2 Smad-specific signaling pathways, the activin/TGF-β pathway that uses type I receptors ACVR1B/TGFβR1/ACVR1C to activate Smad 2/3, or the BMP pathway that uses type I receptors ACVR1/BMPR1A/BMPR1B to activate Smad 1/5/9 (20) (Fig. 1D). Previous studies have shown that AMH appears to signal only through the BMP pathway, and importantly, ACVR1/BMPR1A/BMPR1B are expressed in the same tissues as AMHR2 (22-25). Thus, AMH most prominently signals using a combination of AMHR2 and ACVR1/BMPR1A/BMPR1B to phosphorylate and activate Smad 1/5/9 of the BMP pathway, and not Smad 2/3 of the activin/TGFβ pathway.

**Figure 1.**
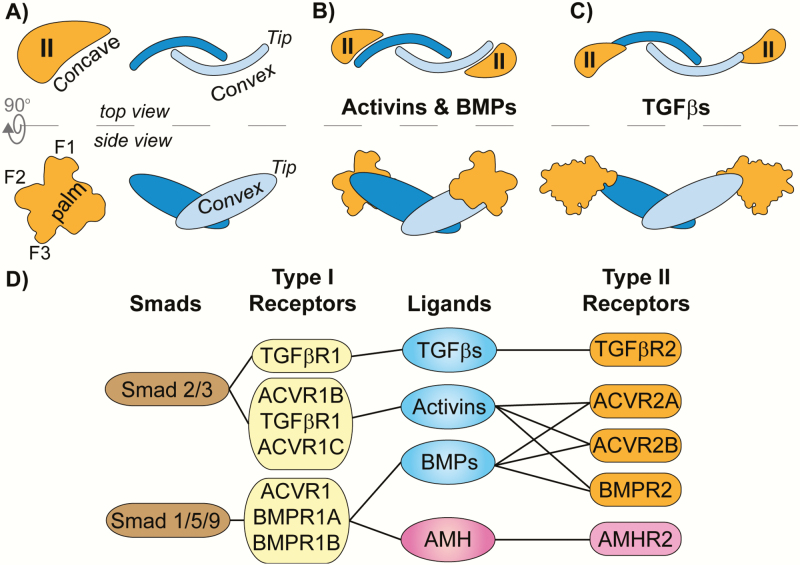
**Type II receptor assembly and signaling paradigms.** (A-C) Top and side view schematics of type II receptors (orange) and ligand dimers (blue) with structural features labeled. Fingers 1 through 3 of the receptor are labeled as F1, F2, and F3 respectively. (B) Activins and BMPs assemble their type II receptors on the concave region of the ligand dimer using the palm of the receptor. (C) TGF-βs assemble their type II receptors on the tip of the ligand dimer using the base of finger 1 (F1). (D) Receptor and Smad preferences of the TGF-β family ligand subclasses. Ligands are connected by lines to the receptors they bind and signal through. Smad proteins are connected by lines to their respective type I receptors which activate them.

Receptors within the TGF-β family have a single extracellular ligand binding domain (ECD, ~13 kDa) which all adopt a similar hand-shaped structure termed the 3-finger toxin fold (20, 21). A series of 3 conserved anti-parallel beta sheets at the core of the receptor form fingers 2 and 3, whereas the size and position of finger 1 varies between receptors (Fig. 2A). The activin and BMP classes use the type II receptors ACVR2A, ACVR2B, and BMPR2, whereas the TGF-β class ligands use TGFβR2 (Fig. 1D). Despite having similar folds, structural studies of the type II receptors in complex with various ligands have revealed 2 general binding schemes. For the activin and BMP classes, the type II receptors bind to the convex surface of the ligand using the concave surface of the receptor core termed the palm (Figs. 1A and B). Within this palm, a cluster of 3 aromatic residues (Tyr or Phe, Trp, Phe), termed the hydrophobic triad, have been shown to be critical for the high-affinity (nM) interactions with the ligands (26-28). Alternatively, ligands of the TGF-β class bind their type II receptor in a completely different mode where receptor binding is shifted toward the distal tips of the ligand (Fig. 1C). Furthermore, the positioning of TGFβR2 is rotated relative to the activin/BMP mode of type II receptor binding. As a result, TGFβR2 mainly engages its ligands using the base of finger 1 and also does not have the hydrophobic triad (29).

**Figure 2.**
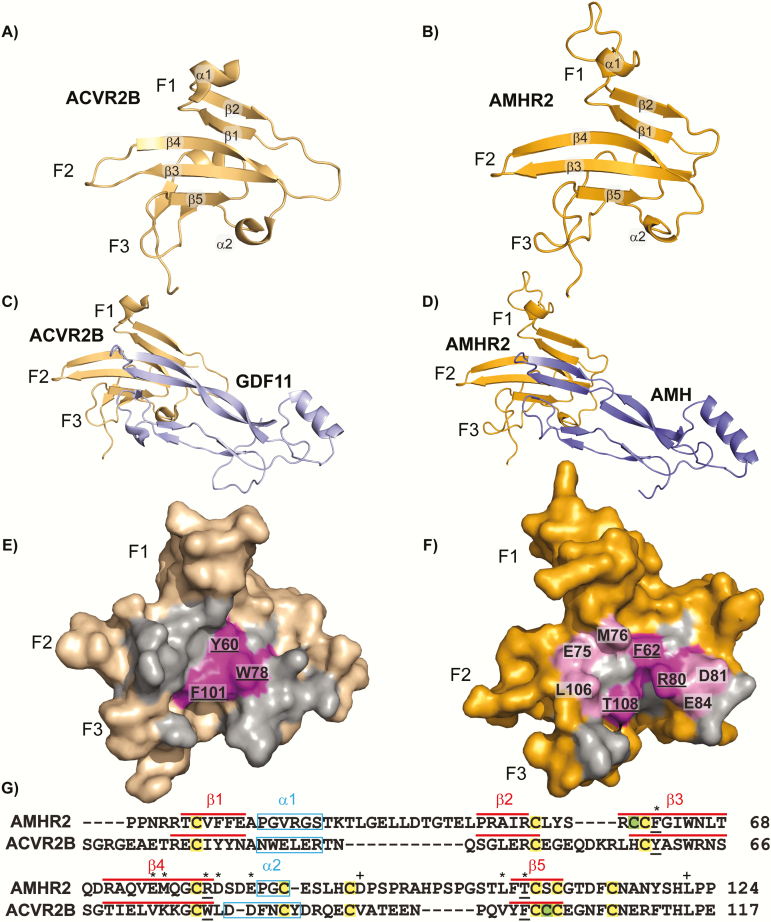
**Model and sequence alignments of ACVR2B and AMHR2.** (A) Structure of ACVR2B-ECD (wheat) in cartoon representation. (B) Swiss-MODEL of AMHR2-ECD (orange) in cartoon representation. Secondary structure elements are labeled accordingly (α-alpha helix, β-beta sheet). (C) Structure of ACVR2B-ECD (wheat) and GDF11 mature (pale blue) in complex from ternary complex (PDBID:6MAC) in cartoon representation. (D) Swiss-MODEL of AMHR2-ECD residues 21–126 (orange) and AMH mature residues 458 through 560 (blue) in complex and in cartoon representation. (E) Surface representation of ACVR2B-ECD and (F) AMHR2-ECD model with all residues within 5Å of the respective ligand highlighted in gray and residues matching the hydrophobic triad of ACVR2B residues in magenta, labeled with underline. Residues used in experiments are highlighted light pink and labeled. Fingers 1, 2, and 3, of the receptors are labeled as F1, F2, and F3, respectively. (G) Sequence alignment of AMHR2 and ACVR2B with residue number indicated to right. Residues used for mutational analysis are labeled with an asterisk. Residues used as controls for mutational analysis are labeled with a plus sign. Residues underlined are part of the hydrophobic triad. Predicted secondary structure is denoted (α-helix in light blue box, β-sheet in red line). Conserved cysteines (C) are highlighted yellow, whereas unique cysteines are highlighted green.

Although structural studies have illuminated how several of the TGF-β family ligands interact with their cognate type II receptors, little is known about how AMH interacts with AMHR2, which has evolved a unique interaction that is highly specific, differing from the type II receptors of the activin/BMP class. Previous studies have done preliminary investigations of the binding between AMH and AMHR2 (30,31). Specifically, a previous study used SPOT array analysis and peptides of AMHR2 ECD to investigate this interaction and suggested that AMH binds AMHR2 different from that of TGF-β, activin, and BMP (31). In this study, we sought to further test this hypothesis by using previous structural information and site-directed mutagenesis to help identify individual AMHR2 residues that are important for AMH signaling and binding.

## Materials and Methods

### Structural modeling and sequence analysis

Amino acid sequence alignments of the human genes were initially completed using Clustal Omega (32). Gaps were adjusted in the sequence alignment based on secondary structure predictions from SABLE (33). All structural alignments with known crystal structures as the reference and all figures with structures or models were generated using PyMOL (34). Structural models of AMH (UniProt ID P03971) and AMHR2 (UniProt ID Q16671) were generated using Swiss-MODEL (35).

### Luciferase assays

All luciferase assays were conducted using transfected HEK-293T cells (ATCC Cat# CRL-3216, RRID:CVCL_0063) (36). Cells were seeded at 20 000 cells per well in growth medium in a 96-well format on poly-D-lysine coated plates (catalog no. 655940, Greiner BioOne GmbH). Cells were grown at 37°C with 5% CO_2_ until 70% to 80% confluence. Cells were then transfected with a total of 100 ng of DNA using TransIT-LT1 Reagent (MIR 2300 Mirus Bio LLC) with the DNA of each BRE (10 ng), AMHR2 (25 ng), and the appropriate type I receptor (10 ng) diluted in OPTI-MEM reduced serum media (31985-070 Gibco, Life Technologies) according to manufacturers’ instructions. Additional pcDNA3 empty vector was added to adjust the DNA to 100 ng per well. 4xBRE plasmid in the pE1b-luc vector backbone was gifted by Joan Massagué (37). The AMHR2 plasmid was gifted by Margrit Urbanek and contained full length (FL) human AMHR2 with a C-terminal 6x myc tag. Type I receptor plasmids were purchased through commercially available sources: human ACVR1 in pcDNA3 vector backbone (plasmid no. 80870, Addgene) and human BMPR1A in pcDNA3 vector backbone (plasmid no. 80873, Addgene). Mouse BMPR1B in pcDNA3 vector backbone was gifted by Dan Bernard. At 24 hours posttransfection, media was replaced with 100 μL serum-free (SF) medium with or without recombinant human AMH mature (rhAMH) protein (LR-MIS mature) (38, 39). Cells were treated with 1 nM rhAMH (purification described in the following section) during luciferase assay development and testing of AMHR2 mutations. For generation of the EC_50_, media was swapped with different concentrations of rhAMH, from 20 nM to 9.8 pM by 2-fold dilutions in SF medium. At 24 hours after media replacement, cells were lysed with 20 μL of 1X passive lysis buffer (catalog no. E1941, Promega) on a plate shaker (900 rpm, 20 minutes, 20°C) then transferred to black and white 96-well plates. Next, 40 μL of Luciferase Assay Reagent (LAR) (catalog no. E1501, Promega) were added to each well, and firefly luciferase luminescence was measured using the Synergy H1 Hybrid Plate Reader (BioTek). All experiments were conducted independently at least 3 times with all data points being done in triplicate per plate. The EC_50_ was calculated using nonlinear regression with variable slope using GraphPad Prism version 5 software.

### Western analysis

Expression of human AMHR2-FL wild-type (WT) and mutant constructs was confirmed by anti-myc Western blotting. Briefly, HEK-293T cells in a 6-well format were seeded (600 000 cells/well) and allowed to grow at 37°C with 5% CO_2_ until ~80% confluence. Cells were transfected with 2.5 μg of either WT or mutant AMHR2-FL DNA using 7.5 μg of PEI diluted in Opti-MEM medium. Media was swapped with 2 mL SF medium 24 hours posttransfection. At 48 hours posttransfection, cells were lysed with 200 μL of 1X passive lysis buffer (900 rpm, 20 minutes, 20°C) then centrifuged to pellet cell debris. Anti-myc (9E10, catalog no. CRL-1729, ATCC, RRID:AB_10573245) (40). Western blots were conducted using a 15% SDS-PAGE gel with 20 μL of cell lysate of each WT or mutant sample. Western blots were developed using the SuperSignal West Pico detection reagent (Thermo Fisher) per manufacturer instructions and detected using a C-DiGit blot scanner (LI-COR).

### Quantitative real-time PCR of HEK-293T cells

mRNA expression levels of native FL human type I receptors ACVR1, BMPR1A, and BMPR1B and FL human type II receptor AMHR2 in HEK-293T cells used for luciferases were evaluated by quantitative real-time PCR (qRT-PCR). Briefly, HEK-293T cells in a 6-well format were seeded (600 000 cells/well) and allowed to grow at 37°C with 5% CO_2_ until ~80% confluence. Cells were transfected with a total 2.5 μg of pcDNA3 empty vector DNA using 7.5 μg of PEI diluted in Opti-MEM medium. Media was swapped with 2 mL SF medium 24 hours posttransfection. At 48 hours posttransfection, cells were processed using the RNeasy kit (catalog no. 74104, Qiagen) per manufacturers protocol to extract mRNA. cDNA was prepared using iScript^TM^ Reverse Transcriptase Supermix (catalog no. 1708840, Bio-Rad) per the manufacturer’s protocol. Expression levels were evaluated by qPCR using iTaq Universal SYBR® Green Supermix (catalog no. 1725121, Bio-Rad) and the CFX96 Touch Real-Time PCR Detection System (Bio-Rad). Data points were collected in triplicate. Each well contained a 20 μL reaction consisting of 150 ng of cDNA, 1.25 μM forward primer, 1.25 μM reverse primer, and 10 μL SYBR green per well in a 96-well PCR plate (catalog no. AB0600, Thermo Fisher). 18S levels were used as the housekeeping gene control.

Additionally, the increase of expression levels was evaluated by transfections of either 750 ng of AMHR2 FL or 300 ng type I receptor (alone or in combination depending on condition), using empty vector to fill DNA levels to 2.5 μg total transfected DNA with 7.5 μg of PEI diluted in Opti-MEM medium. Again, media was swapped with 2 mL SF medium 24 hours posttransfection. At 48 hours posttransfection, cells were processed as described to extract mRNA, make cDNA, and run qRT-PCR.

The PCR primers for human genes are as follows: h18S, 5′-AGTCCCTGCCCTTTGTACACA-3′ and 5′-CCGAGGGCC TCACTAAACC-3′; hACVR1, 5′-GAAGGGCTCATCACCACC AAT-3′ and 5′-GAACGGTGGCTTGTAATCCTC-3′; hBMPR1A, 5′-TGGGCCTTGCTGTTAAATTC-3′ and 5′-ATTCTTCCACGATCCCTCCT-3′; hBMPR1B, 5′-ACACCA CAGGGCTTTACTTAT-3′ and 5′-AATTGCTGGTTTGCCTTGAGT-3′; hAMHR2, 5′- AGGCCTGACAGCAGTCCACCA-3′ and 5′-TTGAGGATGGGCCAAGGCAGC-3′.

### rhAMH protein expression and purification

CHO cells were stably transfected with an optimized FL modified human AMH construct (LR-MIS) as previously described (38, 39). Conditioned media was collected every 48 to 72 hours and purified by antibody affinity chromatography using a monoclonal antibody to AMH (6E11, RRID:AB_2802135) as previously described (38, 41, 42). AMH mature was separated from the Prodomain by rapid change to acidic pH conditions (pH 3-5) and purification by C18 reverse-phase chromatography (Sepax) equilibrated in 0.1% TFA, 5% acetonitrile, and eluted with a linear gradient to 0.1% TFA, 95% acetonitrile over 10 column volumes. Pure AMH mature fractions, confirmed by nonreduced and reduced SDS-PAGE, were then immediately pooled and dialyzed into 10 mM HCl at 4°C overnight. Protein was then concentrated and stored at –80°C for future use.

### AMHR2 ECD protein expression and purification

WT or mutant MBP-fused human AMHR2 ECD constructs containing N-terminal 8x His, myc, and MBP tags, an HRV-3C protease cleavage site, followed by the human AMHR2 ECD residues 18 to 124 were cloned into a pcDNA4 backbone containing a rat IL-2 signal sequence. Constructs were transiently transfected in Expi-293T cells (Life Technologies) and expressed for 5 days. Conditioned media was collected by centrifugation and first purified over NTA-Excel resin (GE Lifesciences) equilibrated in 20 mM sodium phosphate pH 7.4, 500 mM NaCl, and 20 mM imidazole. Bound protein was eluted with equilibration buffer containing 500 mM imidazole then applied to an S200 size exclusion column (Pharmacia Biotech) with a running buffer of 20 mM Hepes 7.5, 500 mM NaCl. Fractions containing pure MBP-fused AMHR2 ECD, confirmed by nonreduced and reduced SDS-PAGE, were pooled then concentrated.

### Native PAGE

The rhAMH protein was kept constant at 0.5 μg and mixed at 4°C for 30 minutes with MBP-fused AMHR2 ECD WT or mutant protein at different molar ratios from 1:2 to 1:32 (AMH mature:AMHR2 ECD) by 2-fold increase with receptor in excess. MBP-fused AMHR2 ECD WT or mutant protein was run in a separate lane at the 1:32 ratio, absent of rhAMH, to serve as a control. Native polyacrylamide gels (12%) were run at 120 V for 4.5 hours at 4°C. Gels were then fixed with 40% ethanol and 10% acetic acid for at least 1 hour and washed 3 times in distilled H_2_O. Gels were stained overnight with a working dye solution of 80% colloidal Coomassie Blue and 20% methanol. Selected bands were excised from native PAGE and ran on SDS-PAGE as previously described to identify protein components (27, 43).

### Statistical analysis

All statistics were calculated using GraphPad Prism version 5 software. One-way ANOVA with Bonferroni’s multiple comparison test was used to determine significance in AMHR2 mutant luciferase assay experiments.

## Results

### Sequence and structural comparison of AMHR2 to ACVR2B

Based on previous evidence that AMH signals through Smad 1/5/9 of the BMP pathway and is more sequentially similar to activins and BMPs, we predict that AMH may bind AMHR2 similarly to the activin/BMP type II receptor binding mode (ie, on the convex ligand surface). Although modeling of AMHR2 has been previously reported (31), neither the structure of AMH nor AMHR2 have been experimentally derived and thus the residues involved in signaling have not been characterized. To generate a putative model of the AMH:AMHR2 complex, we first generated models of the individual components based on known structures of similar proteins. A model of AMHR2 was constructed by Swiss-MODEL using the structure (PDB ID 6MAC) of the activin/BMP type II receptor, ACVR2B (Fig. 2A and B) (35). Superposition of the AMHR2 model with ACVR2B, resulted in a root mean square deviation of 0.49 Å with 90 Cαs aligned from the 107 residues in AMHR2 and 95 in ACVR2B. Overall, this result demonstrates that AMHR2 can be generally modeled using ACVR2B to produce the 3-finger toxin fold. However, an extension observed in the tip of finger 1 that is dissimilar to ACVR2B was not modeled with high confidence. Because TGFβR2 also deviates from ACVR2B with respect to finger 1, we also attempted to model AMHR2 using the structure of TGFβR2. Again, we were unable to model most of finger 1 and the overall model was less similar to the model using ACVR2B, suggesting that TGFβR2 does not serve as a good template. For the ligand, a model of AMH was generated using a combination of growth and differentiation factor 5 (GDF5), a BMP class ligand, and GDF11, an activin class ligand, (PDB ID 2BHK and 6MAC, respectively) and maintained the prototypical fold of a TGF-β ligand (root mean square deviation 0.41 Å).

To generate a model of AMHR2 binding to AMH we used the previous structure of ACVR2B bound to GDF11 (PDB ID 6MAC, Fig. 2C) and superimposed the model of AMHR2 with ACVR2B and the model of AMH with GDF11, respectively, and generated a putative binary complex model of AMH:AMHR2 (Fig. 2D). As would be expected, the buried surface areas of the ligand:receptor interface are similar at approximately 700Å (2, 44). From the binary AMH:AMHR2 complex model, we identified residues within the putative ligand binding interface (Fig. 2F). Furthermore, we generated a sequence alignment of the ECD of AMHR2 to the ECD of ACVR2B using Clustal. This sequence alignment was adjusted by arranging gaps in the sequence so that secondary structure elements of the model were aligned with ACVR2B (Fig. 2G). Intriguingly, several residues within AMHR2 were not conserved with the ligand binding epitope of ACVR2B, including 2 of the 3 corresponding hydrophobic triad residues, previously shown to be essential to the ACVR2B ligand binding properties. From this model and sequence alignment, we selected the 3 corresponding hydrophobic triad residues (F62, R80, T108) in AMHR2 and 5 additional residues (E75, M76, D81, E84, L106) within the putative interface and analyzed their contribution to AMH binding and signaling by mutational analysis (Fig. 2E-G). We also selected 2 residues (D93 and L123) on the back side of AMHR2 that are not predicted to be involved in ligand interactions to serve as controls for the mutational analysis (Fig. 2G).

### Optimization of an AMH responsive high-throughput luciferase assay in HEK-293T cells

**Figure 3.**
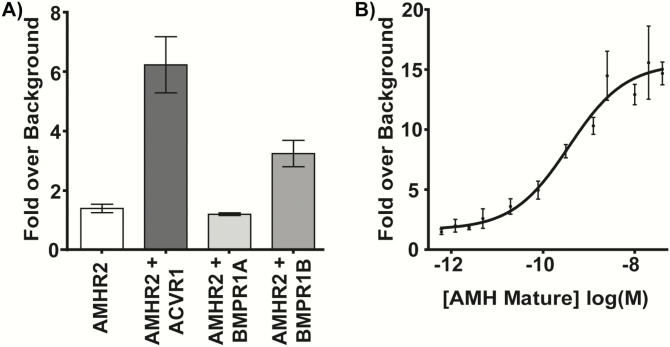
**Development of AMH responsive luciferase assay.** (A) Effects of transfection of 10 ng type I receptors ACVR1, BMPR1A, or BMPR1B. Data are plotted as signaling with 1 nM exogenous rhAMH over no rhAMH (serum-free medium) background. Data represent the average of 3 independent experiments with all data points performed in triplicate per plate. Error bars indicate standard deviation of the fold over background. (B) Representative EC_50_ using exogenous rhAMH in optimized luciferase assay. Data points are signaling fold over background from 2-fold serial dilutions of rhAMH (20 nM-9.8 pM) plotted as log(M) concentrations. Error bars indicate standard deviation of the fold over background for the corresponding rhAMH concentration. Nonlinear regression fit was generated using GraphPad Prism version 5 software with variable slope.

**Figure 4.**
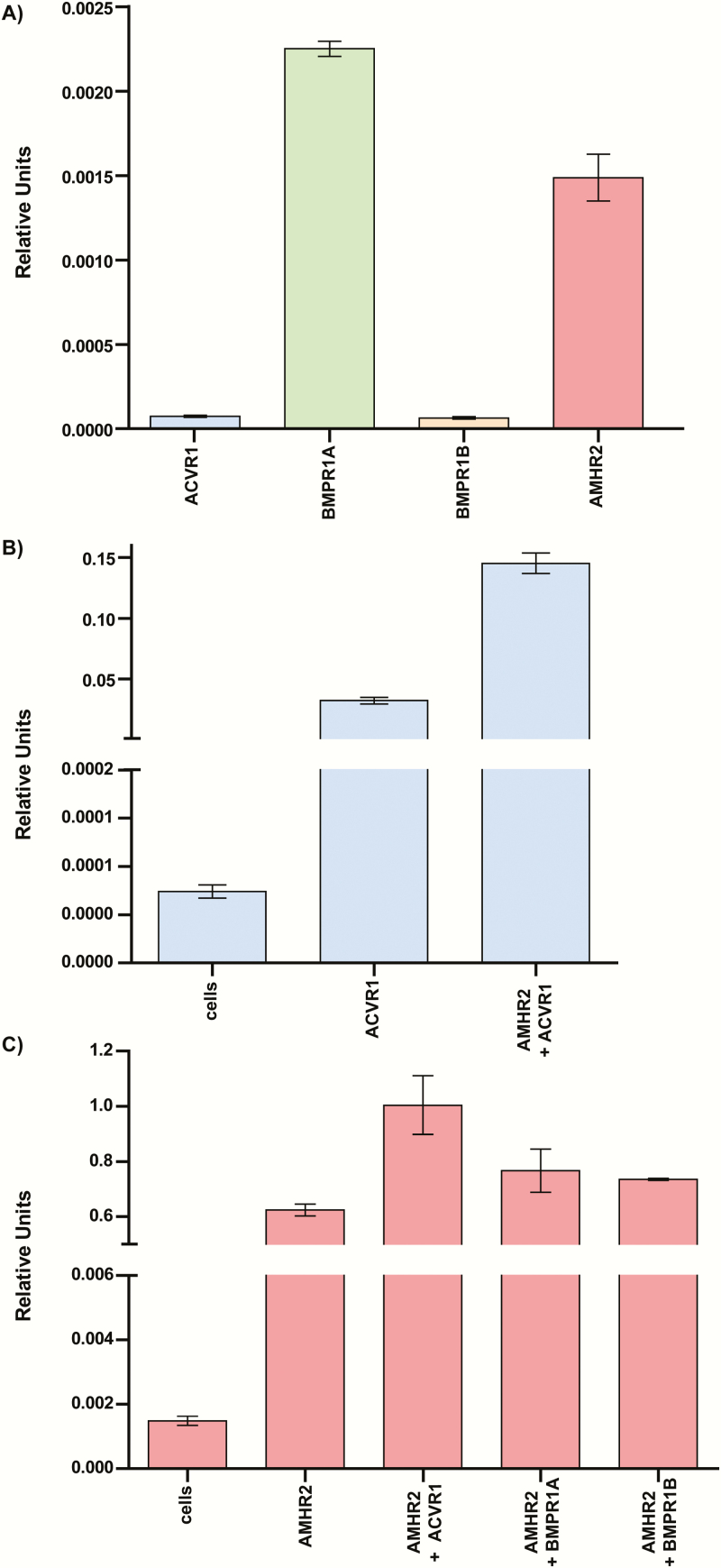
**qRT-PCR of Hek-293T cells with and without transfection.** (A) mRNA levels of ACVR1, BMPR1A, BMPR1B, and AMHR2 in untransfected Hek-293T cells. (B) ACVR1 mRNA levels in untransfected (cells), cells transfected with ACVR1, or cells transfected with ACVR1 and AMHR2. (C) AMHR2 mRNA levels in untransfected (cells), cells transfected with AMHR2, or cells transfected with AMHR2 and either ACVR1, BMPR1A, or BMPR1B. ΔΔCt values are reported as relative units normalized to 18S mRNA levels.

Other groups have previously published AMH-responsive luciferase assays; however, there are none commercially available (14, 45, 46). To test the effects on AMH signaling of mutations in the ECD of AMHR2, we generated our own high-throughput cell-based luciferase assay. Because AMH has been shown to activate downstream effects of the BMP pathway, we transfected the DNA of the BMP responsive luciferase promoter, BRE (47, 48), along with AMHR2, and BMP type I receptors, ACVR1/BMPR1A/BMPR1B, into HEK-293T cells. HEK-293T cells were used because they are easily transfectable, and we have had previous success optimizing SMAD-responsive luciferase assays in these cells (49, 50). We also conducted qRT-PCR of our HEK-293T cells to measure native levels of receptor mRNA and ensure that we were in fact increasing the receptor mRNA levels in excess. We determined that, in our assay, transfection of ACVR1 DNA, in addition to BRE and AMHR2, was essential for obtaining a robust AMH signal with 6- to 20-fold activation over background (Fig. 3). In this assay, we determined that AMH has an EC_50_ of ~0.4 nM, which is consistent with other ligands of the TGF-β family (Fig. 3B) (46, 51, 52). Interestingly, the mRNA levels of native HEK-293T cells showed low levels of all receptor mRNA, with ACVR1 and BMPR1B mRNA being much lower than BMPR1A and AMHR2 levels (Fig. 4A). Furthermore, when we transfected in excess ACVR1 and AMHR2, a significant increase in mRNA levels of these receptors was observed, suggesting that excess receptor is needed for robust luciferase expression (Fig. 4B).

### Residues in AMHR2 required for AMH signaling differ from those in ACVR2B

**Figure 5.**
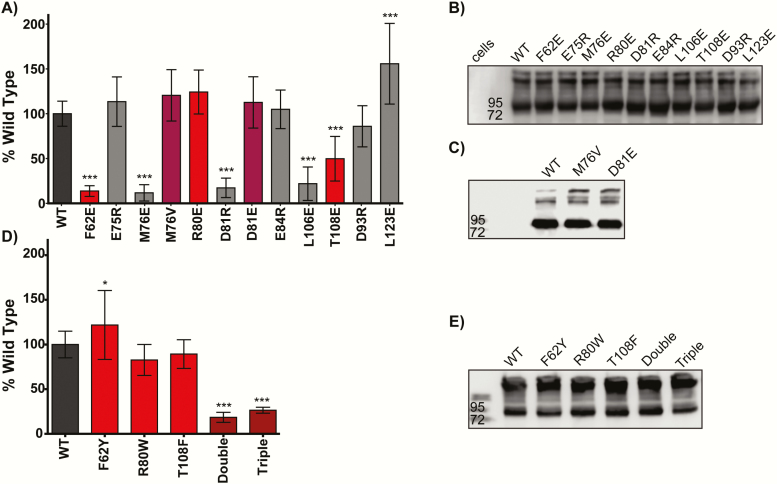
**Analysis of AMHR2 mutants in luciferase assay.** (A) Effects of AMHR2 mutations on AMH signaling plotted as % wild-type with error bars showing standard deviation. Data represent the average of five independent experiments with all data points performed in triplicate per plate. Red bars indicate corresponding residues of the hydrophobic triad. Purple bars represent mutations found in patients with PMDS. (B, C, E) Anti-myc western of AMHR2-FL WT or mutant expression in luciferase assays. (*P ≤ 0.05 and ***P ≤ 0.001, 1-way ANOVA with Bonferroni’s multiple comparison test). (D) Effects of mutating AMHR2 residues to ACVR2B residues on AMH signaling plotted as % wild-type with error bars showing standard deviation. Double indicates mutations of both R80W and T108F and triple indicates the 3 substitutions, F62Y, R80W, and T108F into 1 construct.

To determine which residues from our modeling (Fig. 2) were essential for AMH signaling, we generated point mutations in AMHR2 in the 8 previously mentioned residues using site-directed mutagenesis and tested them in our AMH optimized luciferase assay. We first chose to mutate these residues to either a glutamate (E) or arginine (R) to either introduce a charge to the residue or give the residue an opposite charge. By changing the charge of the residue of interest, our hope was to create the largest negative effect on binding and signaling. Our assays showed that mutation of F62, M76, D81, and L106 nearly abolished signaling in our luciferase assay (*P* ≤ 0.001) (Fig. 5A). However, mutation of E75, R80, E84, and our control, D93, resulted in WT-like signaling (Fig. 5A). Mutation of T108 appeared to decrease signaling by ~50% (*P* ≤ 0.001) (Fig. 5A). Surprisingly, mutation of L123, on the back side of the receptor in our model, appeared to increase AMH signaling by 150% (*P* ≤ 0.001, n = 7) (Fig. 5A). These results indicate that F62, M76, D81, L106, and T108 are essential for receptor-ligand interactions; however, E75, R80, E84, and D93 are likely not essential to AMH signaling. All mutants expressed in levels similar to WT as confirmed by Western blot analysis, indicating that mutation of these residues had no dramatic effect on expression of the receptor (Fig. 5B). Therefore, it is reasonable to assume that the effects seen by these mutations are a result of reduced binding or signaling rather than expression differences.

### Investigation of AMHR2 mutations found in PMDS patients

PMDS mutations have been identified at position M76 and D81 of the receptor (12). Because mutation of both M76 and D81 to a nonconserved substitution dramatically decreased signaling, we next wanted to determine the impact of the 2 conserved PMDS mutations on AMH signaling. Similar to before, we generated the individual M76V and D81E AMHR2 mutations and tested them in the SMAD-dependent luciferase assay. Interestingly, mutation of M76 to valine and D81 to glutamate had no significant effect on AMH signaling in our assay (Fig. 5A). Western blot analysis showed the M76V and D81E were expressed to similar levels as WT receptor (Fig. 5C).

### Can AMH still signal in the presence of the hydrophobic triad?

As mentioned earlier, 2 of the 3 corresponding hydrophobic triad residues in AMHR2 are not conserved. Therefore, we next asked whether introducing the aromatic hydrophobic triad residues into AMHR2 would have an effect on AMH signaling. As such, we mutated the residues F62, R80, and T108 in AMHR2 to the corresponding hydrophobic triad residues of ACVR2B (Tyr, Trp, and Phe, respectively). We found that single substitutions of ACVR2B at these positions had little or no effect on AMH signaling. Interestingly, both the double (R80W, T108F) and triple (F62Y, R80W, T108F) substitutions significantly decreased AMH signaling (Fig. 5D). Again, Western blot analysis was used to rule out changes in expression as a potential confounding factor (Fig. 5E).

### Do mutations that reduce AMH signaling diminish the ability of AMH to bind AMHR2?

**Figure 6.**
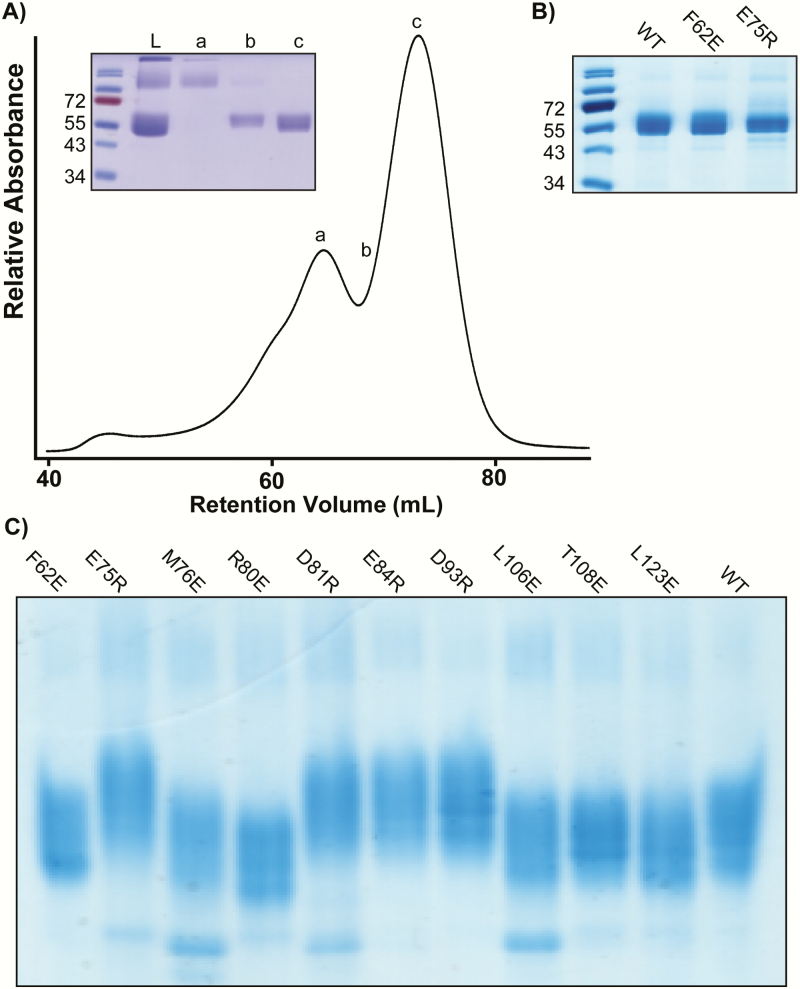
**Purification of recombinant AMHR2 ECD WT and mutants.** (A) Representative trace from size-exclusion chromatography of MBP-fused AMHR2 ECD. Inset SDS-PAGE of peaks (a-c). L denotes sample of material loaded onto column. (B) SDS-PAGE showing MBP-fused AMHR2 ECD from peak “c” used for native PAGE experiments. (C) Native PAGE of WT or mutant MBP-fused AMHR2 ECD. As expected, mutants with residues changed to glutamate (E) migrate faster than WT, whereas residues changed to arginine (R) migrate slower than WT.

**Figure 7.**
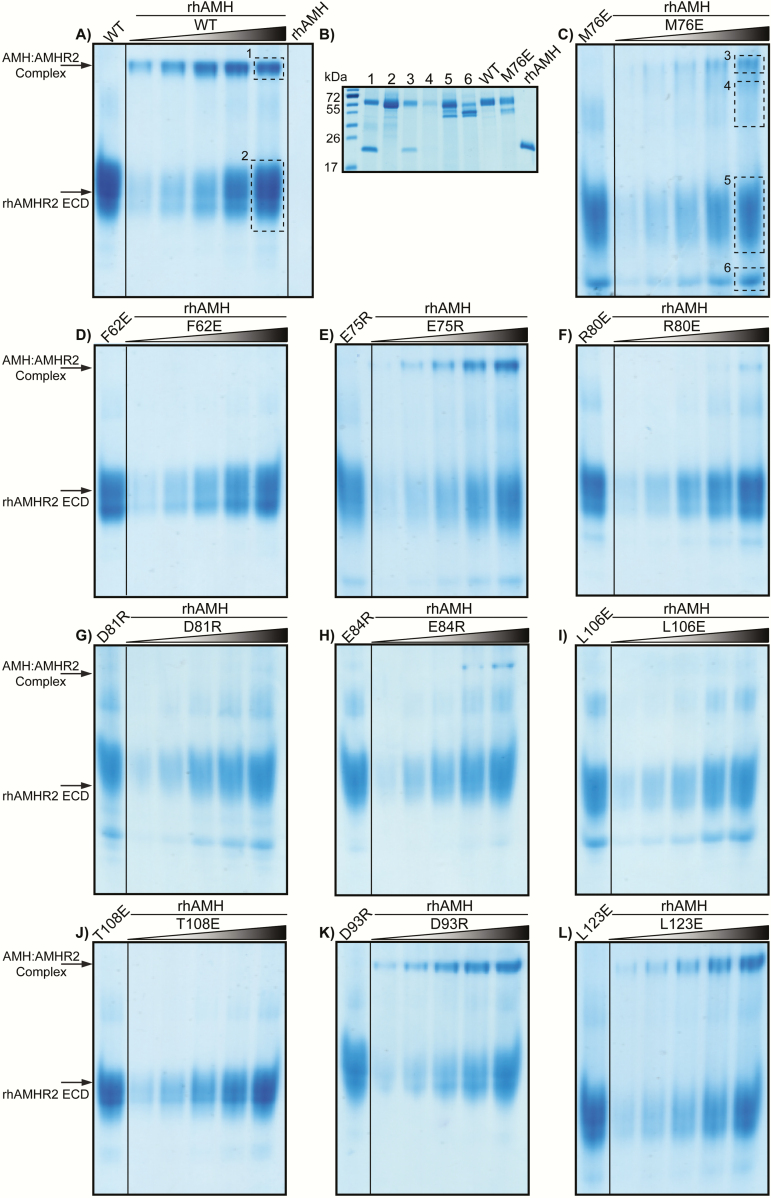
**Native PAGE of MBP-fused AMHR2 ECD with titration of rhAMH.** (A) Complex formation between WT MBP-fused AMHR2 ECD with rhAMH was determined by basic Native PAGE run at 4°C for 4.5 hours at 120 V. First lane of each gel shows the control of receptor alone at the concentration used in the highest ratio of 1:32 during the titration. rhAMH is not visible on basic native PAGE. Complex and MBP-fused AMHR2 ECD alone bands are noted by labeled arrow. rhAMH was incubated with MBP-fused AMHR2 ECD by increasing molar ratios from 1:2 to 1:32 excess receptor. (B) Bands denoted by boxes in (A) and (C) were excised, electro-eluted, and analyzed by SDS-PAGE under reducing conditions C-K). Complex formation test of mutant MBP-fused AMHR2 ECD with rhAMH.

Next, we asked whether the reduction in AMH signaling associated with mutations in AMHR2 were caused by the decreased ability of AMH to bind and form a complex with AMHR2. To answer this question, we first generated recombinant human AMHR2 (rhAMHR2) ECD. Expression of AMHR2 was significantly enhanced when using a fusion protein consisting of an N-terminal His/*myc*/MBP. The MBP fusion was, in fact, essential to produce active AMHR2 ECD, as the same construct without the MBP fusion resulted in only aggregated material. The extracellular domain of AMHR2 consisting of residues 18 through 124 was produced in Expi-293T cells. We next generated recombinant protein of each of the E or R AMHR2 mutants. Once purified (~95%) by size exclusion chromatography 200 and confirmed by SDS-PAGE (Fig. 6A and B), proteins were analyzed for ligand:receptor complex formation by native PAGE. First, the individual receptor fusion proteins (WT or mutant AMHR2) were analyzed by native PAGE (Fig. 6C). Mutations in which a substitution introduced an arginine (R) caused the receptor band to migrate slower than WT, whereas substitutions to glutamate (E) caused the receptor band to migrate faster than WT—consistent with the charge differences of the receptor as separated by native PAGE. Subsequently, we tested the ability of WT or mutant rhAMHR2 ECD to form a complex with rhAMH on native PAGE. Because 2 receptors are expected to bind to 1 AMH dimer, we analyzed complex formation under excess receptor ratios (1:2-1:32 ratios), while keeping rhAMH constant. The first lane of each gel shows WT or mutant rhAMHR2 ECD at the highest ratio used for titrations without rhAMH (Fig. 7). rhAMH does not enter the gel and therefore is not visible in native PAGE (Fig. 7A). Complex bands can be seen for WT, M76E, E75R, R80E, E84R, D93R, and L123E (Fig. 7A, C, E, F, H, K, and L). Mutations of F62E, D81R, L106E, and T108E do not appear to form a complex band up to a ratio of 1:32 excess receptor (Fig. 7D, G, I, and J).

To confirm that our complex bands did indeed contain both rhAMH and rhAMHR2 ECD, we excised the bands observed in native PAGE, electroporated the bands, and analyzed them on SDS-PAGE as previously described (27, 43)(Fig. 7A-C). As such, 2 bands are visible, 1 at ~55 kDa and 1 at ~24 kDa corresponding to rhAMHR2 ECD and rhAMH, respectively.

## Discussion

Even though AMH has been shown to play a vital role in reproductive development and disease, how AMH engages its receptors to potentiate signaling at the molecular levels has not been characterized. Although we have a good understanding of how representative ligands of each TGF-β class engage their receptors, AMH does not fit into any of the already established classes. As such, the results of our study help define the residues of AMHR2 that are important to AMH signaling. Specifically, 4 (F62, M76, D81, L106) of the mutants tested decreased AMH signaling by 75% (Fig. 5A) or more and showed no binary formation on native PAGE (Fig. 7), only 1 of which is in the corresponding hydrophobic triad. Mutation of T108, a corresponding hydrophobic triad residue, decreased AMH signaling by approximately 50% (Fig. 5A) and showed no binary complex formation in native PAGE (Fig. 7). Furthermore, we identified residues that, when mutated, had no significant effect on AMH signaling and were able to form a binary complex, including R80, E75, E84, and D93 (Figs. 5A and 7). Curiously, mutation of L123 to a glutamate resulted in a slight increase in AMH signaling (Fig. 5A). These results show that AMHR2 has a highly modified ligand binding epitope relative to ACVR2B as residues important for ligand binding are not conserved. Furthermore, these results bring to light the broader molecular differences that have evolved within the different TGF-β type II receptors.

As previously mentioned, several mutations in AMH and AMHR2 have identified in patients with PMDS. Two residues were analyzed in this study (M76 and D81) and have been shown to result in the development of PMDS (12). At these positions, we found that mutations to nonconserved changes significantly decreased AMH signaling, whereas more conservative changes to M76 and D81 (M76V and D81E (12)), identified in PMDS patients, had no effect on AMH signaling (Fig. 5A). Although these results highlight the importance of these residues to the interaction with AMH, they also demonstrate that naturally occurring PMDS mutations result in a receptor that is still functional (ie, not misfolded) and expresses similar to WT receptor in HEK-293T cells. Thus, it is interesting to speculate how these mutations contribute to PMDS. It is possible that the developmental process is highly sensitive to changes in AMH signaling activity. It is also possible that these mutations alter receptor localization or receptor stability. Further tests using in vivo models are needed to fully understand the direct effects of these mutations within PMDS.

Surprisingly, although mutation of M76 resulted in a striking reduction in AMH signaling in the BRE luciferase reporter assays (Fig. 5A), M76E still produced a ligand:receptor binary complex on native PAGE as confirmed by gel excision and SDS-PAGE, albeit much weaker than WT (Fig. 7B and C). How this substitution is able to still form complex, but does not signal, is certainly intriguing and will require additional experimentation. Nevertheless, a version of AMHR2 that still binds the ligand, but does not signal, could constitute a useful molecular tool or future therapeutic strategy to neutralize excess AMH signaling.

In our assays, comparing the binding data from the native PAGE analysis with the signaling results has its limitations. For one, the native PAGE analysis uses a fusion of AMHR2 bound to MBP, whereas the luciferase assay uses the full length receptor and includes the type I receptor. It is possible that the type I receptor plays a cooperative role in the stabilization of the signaling complex, similar to TGF-β. This may explain why, in certain cases, we see a reduced affinity for binary complex from R80E compared with WT yet signaling resembles that of WT. It is also possible that, in this particular case, the fusion protein of R80E (ie, MBP) reduces AMH affinity.

**Figure 8.**
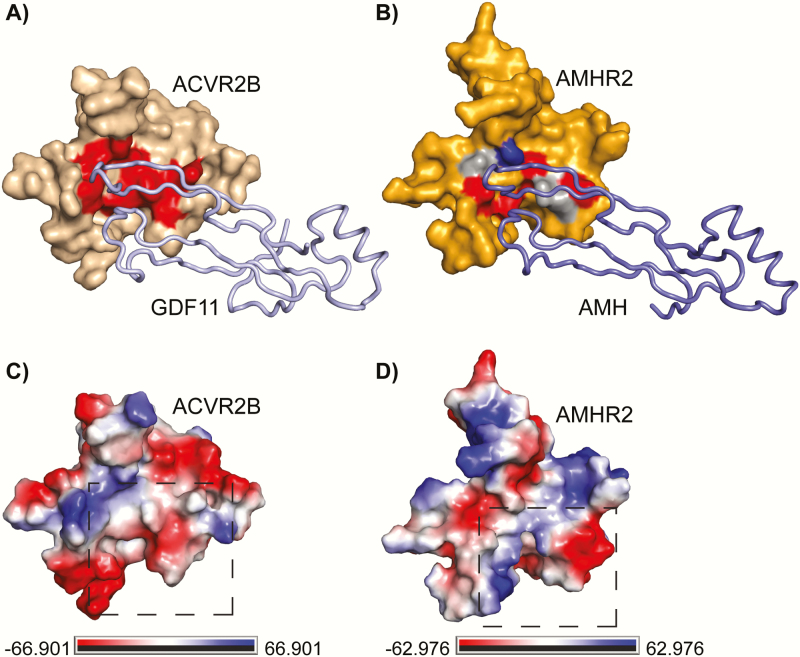
**Overview of residues important for ligand binding.** (A) ACVR2B in wheat surface bound to GDF11 in pale blue ribbon. Residues important for ligand binding are highlighted in red. (B) AMHR2 in orange surface bound to AMH in blue ribbon. Residues important for AMH signaling are highlighted in red, whereas residues that had no effect when mutated are highlighted in gray. M76 is highlighted in royal blue. (C, D) Vacuum electrostatics map of (C) ACVR2B or (D) AMHR2 with positive charge in royal blue, negative charge in red, and hydrophobic in white from –66.901 to 66.901 k_b_t/e_c_ (ACVR2B) or from –62.976 to 62.976 k_b_t/e_c_ (AMHR2).

As mentioned, AMH is the only member of the TGF-β family with its own dedicated type II receptor. How AMH and AMHR2 have evolved such high levels of specificity has yet to be determined. This is particularly interesting from a molecular standpoint since a number of TGF-β family ligands interact with multiple receptors and, vice versa, receptors interact with multiple ligands. Thus, AMH and AMHR2 have evolved molecular interactions that maintain an exquisite interaction. One possibility into how this fidelity is generated is that AMHR2 has a modified ligand binding epitope that only allows AMH binding. Evidence for this might be apparent when comparing the model of AMHR2 to ACVR2B. If we color the AMHR2 model and the structure of ACVR2B using electrostatic potential, clear differences exist between the 2 receptor surfaces near the ligand binding epitope (Fig. 8). It is clear that AMHR2 contains more positively charged (blue) residues in the putative ligand binding site (Fig. 8D) compared with the largely hydrophobic (white) and negatively charged (red) center of ACVR2B (Fig. 8C). Additionally, residues distal to the putative binding pocket are negatively charged (red) in AMHR2 (Fig. 8D) compared with the positively charged (blue) in ACVR2B (Fig. 8C). Furthermore, when we introduced the hydrophobic triad residues found in ACVR2B into AMHR2, single mutations did not drastically affect AMH signaling. This suggests that AMH signaling is less dependent on a hydrophobic core interaction, unlike activin/BMP ligands for which it is essential. Furthermore, when all three residues (F62Y, R80W, and T108F) in the core of AMHR2 are hydrophobic, AMH is no longer able to signal (Fig. 5D). In fact, double mutation of R80W and T108F also resulted in ablation of signaling. Here, the triad is maintained because F62 is already a large hydrophobic sidechain. Thus, it appears ligand specificity is partially generated by the presence or absence of the hydrophobic triad. On the ligand side, A341 of activin A points into the hydrophobic triad, whereas, the corresponding residue in AMH is an isoleucine (I479). Because isoleucine is much larger than alanine, it might be the case where AMH is unable to accommodate the bulky hydrophobic side chains of the triad. Together, these results provide evidence that AMHR2 uses the palm region of the receptor. This is in contrast to the previous analysis of this interface, which showed the palm of the receptor was not important for ligand interactions using AMH-derived peptides to investigate AMHR2 binding (31). Because the AMH epitope is not expected to be linear, the previous peptide study likely does not adequately recapitulate the AMH interaction epitope (31). Thus, although the ligand binding epitope of AMHR2 appears to be in a similar position to ACVR2B, residues at the interface are different, providing specificity for ligand-receptor interactions. It is also possible, because of the structural differences of the receptor, that regions outside the palm of the receptor could also be important for ligand specificity.

Because we expect AMH to look more like an activin/BMP class ligand than a TGF-β class ligand, it is likely that AMH and AMHR2 maintain this fidelity through specific residue interactions. However, it is also possible that larger structural differences in AMHR2 compared with ACVR2B and TGFβR2 also play a role in specificity. One possibility could be a difference in the positioning of the disulfide bonds of the receptor. In general, the pairing of cysteines which form disulfide bonds within these receptors is well conserved. Both AMHR2 and ACVR2B contain 10 cysteines that form 5 disulfide bonds. Interestingly, all the cysteines align with 1 exception (Fig. 2G, green) where 1 of the 3 consecutive cysteines in β-strand 5 (β5) of ACVR2B is now located in β-strand 3 (β3). In our model and the previously published model of AMHR2 the position of the altered disulfide bond would connect the base of finger 2 to the loop that connects fingers 2 and 3 (31). All other receptors instead form a disulfide bond that connects the base of finger 3 with this loop. This difference would certainly make a larger impact in the overall structure and positions of the corresponding loops that are predicted to be near the ligand interface. Further comparisons to TGFβR2 also highlight structural differences that could impact specificity. Although finger 1 of AMHR2 has a fingertip extension similar to TGFβR2, an additional disulfide bond in TGFβR2 tethers finger 1 into a conformation important for ligand binding. However, it is unlikely that AMHR2 folds similarly to TGFβR2, as Swiss-MODEL could not define this region using TGFβR2 as a template, and AMHR2 does not contain a disulfide bond similar to TGFβR2. Therefore, it is possible that structural changes in AMHR2 are responsible for specificity; however, further structural information of the AMH:AMHR2 binary complex is needed to fully understand this specificity.

In summary, our studies show that AMH likely binds AMHR2 using a similar binding location to ligands that bind ACVR2B, as residues mutated within the palm of AMHR2 were shown to negatively impact AMH signaling (Fig. 8). However, AMHR2 exhibits a number of features that are unique, including significant differences in the residues that, in ACVR2B, compose the hydrophobic triad. Additional investigation of residues within AMH important for AMHR2 binding will help to further elucidate this molecular interaction. Therefore, although the structure of AMH and AMHR2 still remains unknown, it is clear that AMH likely engages AMHR2 with similar, but not identical, mechanisms used by activin and BMP ligands binding their type II receptors.

## Abbreviations

AMH: anti-Müllerian hormone
AMHR2: anti-Müllerian hormone receptor type II
BMP: bone morphogenetic protein
ECD: extracellular ligand binding domain
FL: full length
GDF: growth and differentiation factor
MIS: Müllerian inhibiting substance
PCOS: polycystic ovarian syndrome
PMDS: persistent Müllerian duct syndrome
qRT-PCR: quantitative real-time PCR
rhAMH: recombinant human AMH mature
rhAMHR2: recombinant human AMHR2
SF: serum-free
WT: wild-type.

## Additional Information

***Disclosure Summary:*** T.B.T. consults for Acceleron Pharma. The remaining authors have nothing to declare.

***Data Availability:*** All data generated or analyzed during this study are included in this published article or in the data repositories listed in References.
